# A multicenter phase II study of everolimus in patients with progressive unresectable adenoid cystic carcinoma

**DOI:** 10.1186/1471-2407-14-795

**Published:** 2014-11-03

**Authors:** Dong-Wan Kim, Do-Youn Oh, Seong Hoon Shin, Jin Hyoung Kang, Byoung Chul Cho, Joo-Seop Chung, HyeJin Kim, Keon Uk Park, Jung Hye Kwon, Ji-Youn Han, Mi-Jung Kim, Yung-Jue Bang

**Affiliations:** Department of Internal Medicine, Seoul National University Hospital, 101 Daehak-ro, Jongno-gu, Seoul, 110-744 Republic of Korea; Cancer Research Institute, Seoul National University College of Medicine, Seoul, Republic of Korea; Kosin University Gospel Hospital, Busan, Republic of Korea; Catholic University Seoul St. Mary’s Hospital, Seoul, Republic of Korea; Yonsei Cancer Center, Seoul, Republic of Korea; Pusan National University Hospital, Seoul, Republic of Korea; Seoul Veterans Hospital, Seoul, Republic of Korea; Keimyung University Dongsan Hospital, Daegu, Republic of Korea; Hallym University Medical Center, Seoul, Republic of Korea; National Cancer Center, Gyeonggi-do, Republic of Korea

**Keywords:** Adenoid cystic carcinoma, Everolimus, RAD001, Clinical trial

## Abstract

**Background:**

The aim of this study was to examine the efficacy and safety of everolimus in patients with progressive unresectable adenoid cystic carcinoma (ACC).

**Methods:**

Histologically confirmed ACC patients with documented disease progression within 12 months prior to the study entry were eligible. Everolimus was given at a dose of 10 mg daily until progression or occurrence of unacceptable toxicities. The primary endpoint was a 4-month progression-free survival (PFS).

**Results:**

A total of 34 patients were enrolled. The 4-month PFS probability was 65.5% (95% one-sided confidence interval [CI], 47.7 to infinity). Median PFS duration was 11.2 months (95% CI, 3.6 to 15.8). Complete or partial response was not achieved. Twenty-seven (79.4%, 95% CI, 63.2 to 89.6) patients showed stable disease (SD). Tumor shrinkage within SD criteria was observed in 15 patients (44.1%) and SD lasting 6 months was observed in 13 patients (38.2%). Four patients had disease progression. Among the 18 patients with both pre- and post-treatment (at 8 weeks) FDG-PET scans available, 8 patients (44.4%) showed a partial metabolic response, defined as a ≥25% reduction in maximum standardized uptake values (SUVmax). The most common adverse events were stomatitis, anemia, asthenia, and leukopenia. No unexpected everolimus related toxicities were reported.

**Conclusions:**

Everolimus showed promising efficacy and good tolerability in progressive unresectable ACC.

**Trial registration:**

ClinicalTrials.gov identifier, NCT01152840

## Background

Adenoid cystic carcinoma (ACC) is a rare epithelial malignancy that arises in secretory glands, particularly in the salivary glands. Although the histologic appearance of ACC is low grade, management of this malignancy is a distinct therapeutic challenge because of its tendency for perineural involvement and potential for distant metastasis [1]. The natural course of metastatic disease is relatively indolent; however, most patients with metastatic disease ultimately die from their cancer [2]. Therefore, a more effective treatment strategy for unresectable disease is definitely required.

Cytotoxic chemotherapies have been evaluated for advanced ACC in a numbers of clinical trials of. A systematic review of systemic therapy for advanced ACC reported that objective responses to any cytotoxic agent or regimen were very infrequent, whereas stabilization of disease was observed more commonly [3]. Rates of disease stabilization need to be interpreted with caution in an indolent cancer; however, disease stabilization may be only a marker of antitumor activity. Assessment of disease stabilization is more useful if disease progression is documented before the study entry.

Recently, a series of targeted agents were tested for the treatment of advanced ACC. However, no study has focused on the phosphatidylinositol 3-kinase (PI3K)-Akt-mammalian target of rapamycin (mTOR) pathway in ACC. According to the Younes et al. [4], ACC cell lines exhibited increased phosphorylated Akt activity when stimulated with epidermal growth factor (EGF). And, when treated with epidermal growth factor receptor (EGFR)/vascular endothelial growth factor receptor (VEGFR) tyrosine kinase dual inhibitor, the phosphorylated form of Akt decreased even though the total level of Akt is remained unchanged. Of note, an ACC patient had clinical response to everolimus in a phase I study [5]. Therefore, we performed this phase II study to evaluate the efficacy of everolimus in advanced ACC. We required documented evidence of disease progression to exclude those patients with stable disease due to intrinsically slow growth rate.

## Methods

This open-label, multicenter, phase II, single arm study (ClinicalTrials.gov identifier, NCT01152840) was conducted at 9 hospitals in Korea. The study was conducted in compliance with Good Clinical Practice, guidelines of the International Conference on Harmonisation, and the Declaration of Helsinki, and approved by the local institutional review boards (IRBs) of Seoul National University Hospital, Kosin University Gospel Hospital, Catholic University Seoul St. Mary’s Hospital, Yonsei Cancer Center, Pusan National University Hospital, Seoul Veterans Hospital, Keimyung University Dongsan Hospital, Hallym University Medical Center, and National Cancer Center in Korea. Written informed consent was required from all patients before participation.

### Study population

Adult patients with histological evidence of advanced or metastatic adenoid cystic carcinoma were eligible for this study. Evidence of disease progression according to the Response Evaluation Criteria in Solid Tumors (RECIST) criteria (version 1.0) [6] must be documented by CT or MRI scans taken within 12 months prior to the baseline evaluation and compared to a previous scan taken at any time in the past. Previous treatment with chemotherapy, radiation therapy or surgery were permitted providing that toxicity had resolved to ≤ grade 1 at study entry and that last treatment was at least 4 weeks prior to baseline assessment. Patients were required to have measurable lesions according to the RECIST criteria (version 1.0), a WHO performance status of 0-1 [7], and adequate hematologic, renal, and hepatic function. Patients with previous active or passive immunotherapy, intestinal obstruction or impending obstruction, recent active upper gastrointestinal bleeding, history of another malignant disease within the past 5 years (except for curatively treated basal cell carcinoma of skin and cervical carcinoma in situ), medically uncontrolled systemic disease, interstitial pneumonia or diffuse symptomatic pulmonary fibrosis were not eligible. Pregnant or lactating women were excluded.

### Treatment and evaluation

Patients received 10 mg of daily oral everolimus and one cycle was comprised of 28 days. Treatment was continued until disease progression, unacceptable toxicity, or consent withdrawal. Concomitant anticancer agents other than everolimus were not allowed during the study. Response, based on RECIST criteria (version 1.0), was evaluated every 8 weeks until progression was observed, and survival status was assessed every 12 weeks after the end of treatment visit. Metabolic response was assessed by ^18^F-fluorodeoxyglucose (FDG) positron emission tomography (PET) scan in selected cases. FDG-PET scan was performed at screening and at 8 weeks of treatment. Metabolic response was evaluated as described in a previous study [8]. Briefly, a metabolic CR was defined as a complete resolution of FDG uptake within the tumor so that it was indistinguishable from surrounding normal tissue. A metabolic PR was defined as a reduction of ≥25% in tumor maximum standardized uptake values (SUVs) of FDG uptake. An ≥25% increase in tumor maximum SUVs or the appearance of new FDG uptake in another region was defined as metabolic progressive disease (PD). Metabolic stable disease (SD) was defined as an increased in the tumor SUV of <25% or a decrease of <25%. Safety assessments, including history taking, physical examination, and laboratory evaluation, were carried out at baseline and at the end of each cycle. Adverse events was monitored and recorded according to the National Cancer Institute Common Terminology Criteria for Adverse Events (NCI-CTCAE) version 3.0 during the treatment phase and for 28 days after the final dose of the study medications. Only serious adverse events were reported during the 28 days after the final dose of the study medications in the post-study treatment phase. Dose modifications or delays in study drug administration were allowed as per protocol. When the study medication was delayed, all the evaluations, including tumor evaluation, adhered to the original schedule. Reasons for changes in dose or delays in administration, measures and outcome were recorded in the case report form. The patient was considered to be an early drop-out due to toxicity if administration of study medications was either delayed for ≥3 weeks or discontinued due to toxicity.

### Statistical methods

The primary efficacy end point was progression-free survival (PFS) at 4 months. Patients who received at least one dose of everolimus were included in the intent-to-treat (ITT) population. All efficacy and safety analyses were performed on the ITT population. The hypothesis of this study was that the 4-month PFS rate would be ≥65%. The study design required a minimum of 29 patients to test the null hypothesis that the true proportion of patients who remained progression-free at 4 months from study entry is at most 50%, with 80% power to detect a 4-months PFS proportion of 65%, with a one-sided hypothesis test and an Type I error of 0.05. This sample size was based on the assumptions that patient survival followed an exponential distribution and that no patients would be lost to follow-up. Assuming a dropout rate of 15%, the required number of patients was 33. The secondary endpoints included objective response rate, disease control rate, duration of responses, and length of overall survival (OS) after initiation of the study medication. The median PFS and OS and their confidence intervals (CIs) were calculated using Kaplan-Meier method [9]. P-value for the one-sided hypothesis test was calculated using a normal distribution approximation of the survival rate with its standard error. The frequency and severity of adverse events (AEs) were analyzed. Statistical analysis was conducted using STATA version 12.0 (StataCorp LP, College Station, TX, USA).

## Results

### Patients

From July 2008 through October 2010, 34 patients were enrolled. All those patients received at least one dose of everolimus (ITT population) and were included in the efficacy and safety analyses. The patients’ baseline characteristics are summarized in Table 1. The salivary gland was the most common primary site, and the other primary sites included the paranasal sinus, oral cavity, nasal cavity, larynx, lung, and the Bartholin gland. The median interval from initial diagnosis of ACC to initiation of study treatment was 4.4 years. The most common metastatic site was lung. At the censoring date (Mar 25, 2013), 20 progression events and 20 deaths had occurred, and the median length of follow-up was 19.8 months (range 2.5 to 54.2).

**Table 1:** **Baseline characteristics of patients** (**intention**-**to**-**treat population**, ***N*** = **34**)

Characteristic	
**Age** - **year**	
Median	54
Range	27-73
**Gender** – ***N*** **(%)**	
Male	18 (52.9)
Female	16 (47.1)
**Duration of disease** - **year**	
(from initial diagnosis to study enrollment)	
Median (Mean)	4.4 (6.0)
Range	0.5-22.0
**Primary site** – ***N*** **(%)**	
Salivary gland	13 (38.2)
Paranasal sinus	8 (23.5)
Oral cavity/oropharynx	6 (17.6)
Nasal cavity/nasopharynx	2 (5.9)
Larynx	2 (5.9)
Lung	1 (2.9)
Bartholin gland	1 (2.9)
Unknown	1 (2.9)
**Site of metastasis** – ***N*** **(%)**	
Lung	32 (94.1)
Bone	8 (23.5)
Non-regional lymph nodes	5 (14.7)
Liver	3 (8.8)
Soft tissue	3 (8.8)
Pleura	2 (5.9)
Kidney	2 (5.9)
Adrenal gland	1 (2.9)
Peritoneum	1 (2.9)
Brain	1 (2.9)
Spleen	1 (2.9)
Eyeball	1 (2.9)

### Efficacy

**Figure 1:**
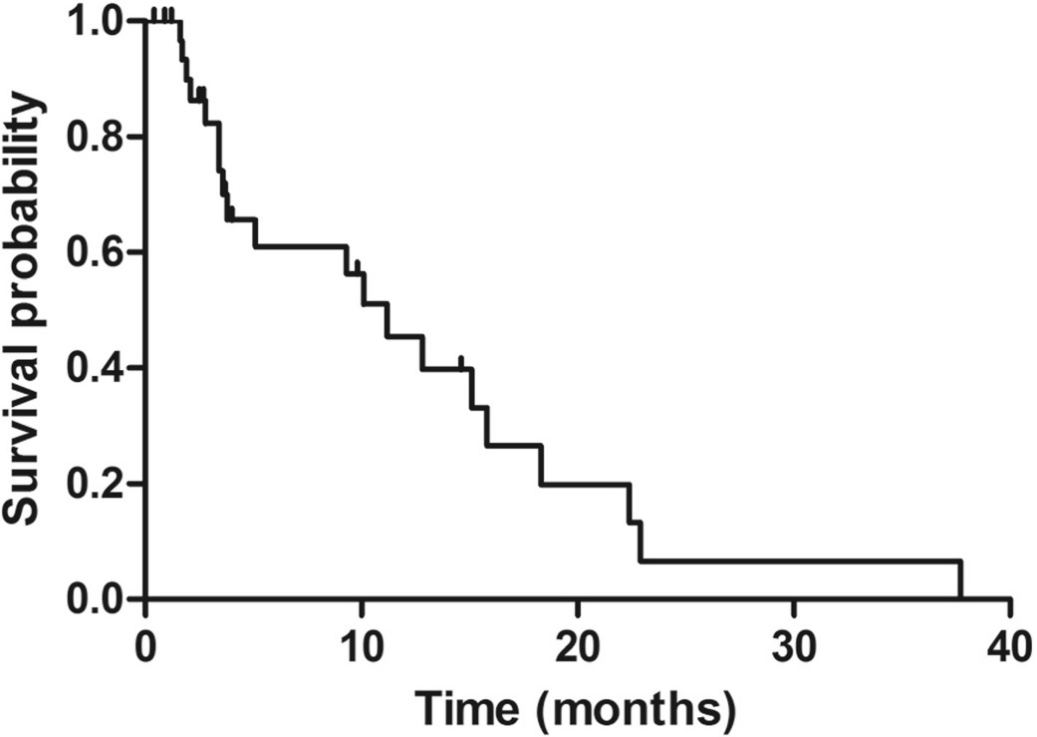
**Progression free survival** **(intention-** **to** **-treat population,** ***N =*** **34).**

**Figure 2:**
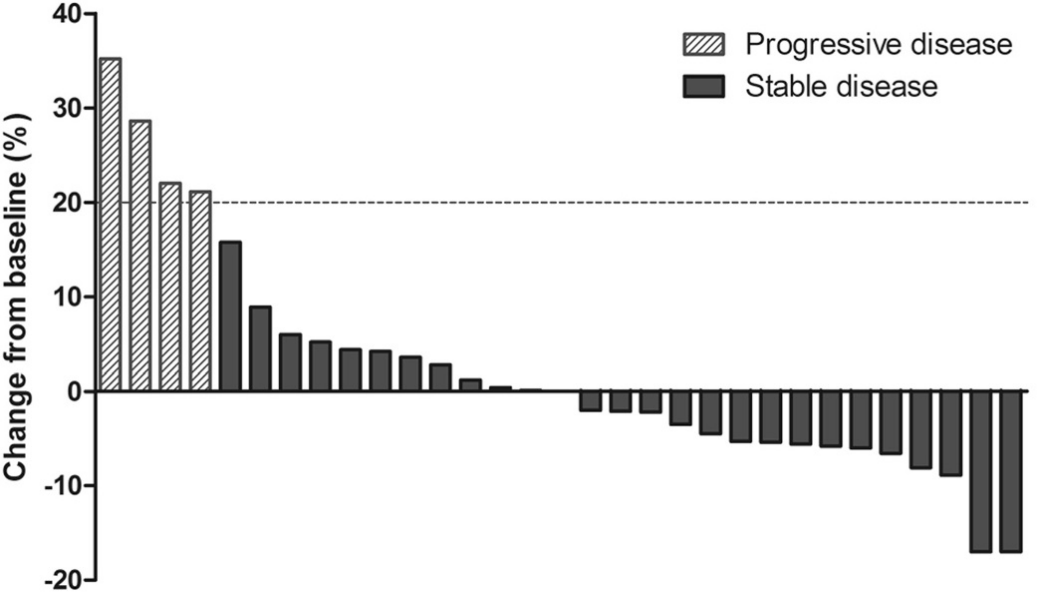
**Best percent changes in tumor size by patients** **(response evaluable patients,** ***N =*** **31).**

**Figure 3:**
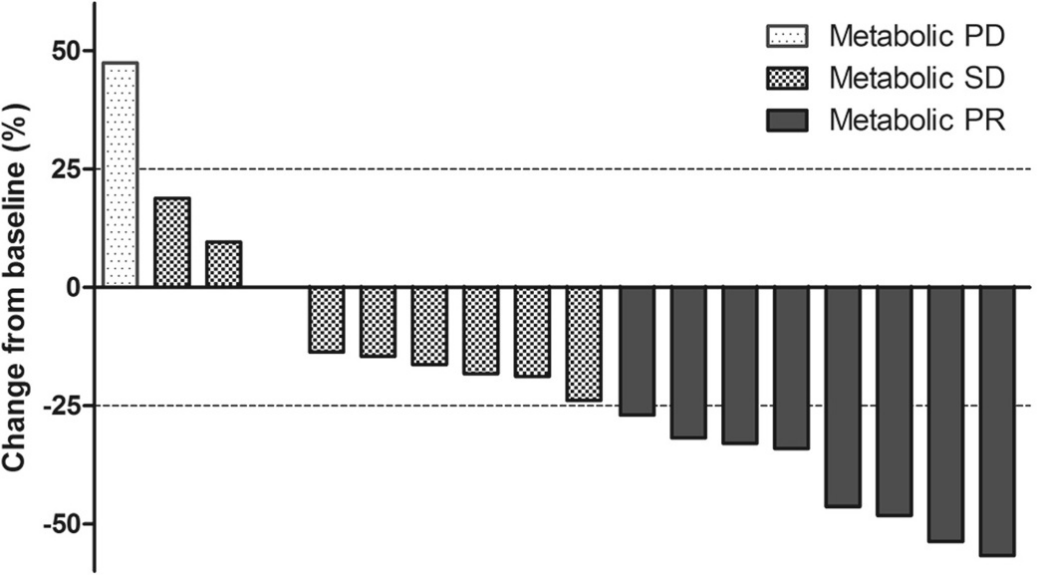
**Percent changes in maximum standardized uptake values** **(SUVmax)** **after 2 cycles of everolimus treatment** **(FDG-** **PET evaluable patients,** ***N*** **=** **18).**

The 4-months PFS probability was 65.5% (95% one-sided CI, 47.7 to infinity) but did not differ significantly from the null hypothesis of a 4-months PFS rate ≤50% (*P* = 0.076). Median PFS duration was 11.2 months (95% CI, 3.6 to15.8) (Figure 1). No patient achieved CR or PR. Twenty-seven (79.4%) patients had SD (95% CI, 63.2 to 89.6). Tumor shrinkage within the SD criteria was observed in 15 (44.1%) patients (Figure 2) and SD >6 months was observed in 13 patients. Four patients had PD (Table 2). Pre-treatment and post-treatment (after 8 weeks) FDG-PET scan was available for 18 patients. All these 18 patients had SD based on RECIST criteria. Among them, the scans indicated metabolic PR in 8 patients, metabolic SD in 9 patients, and metabolic PD in one patient (Figure 3). The median PFS duration of the 8 patients with metabolic PR was numerically longer than that that of the 10 patients with metabolic SD or PD (15.1 versus 3.8 months). The median OS was 23.7 months (95% CI, 6.8 to 40.6).

**Table 2:** **Best overall responses** (***N*** = **34**, **intention**-**to**-**treat population**)

Response, ***N*** (%)	
Complete Response (CR)	0 (0.0)
Partial Response (PR)	0 (0.0)
Stable Disease (SD)	27 (79.4)
Progressive Disease (PD)	4 (11.8)
Not evaluable	3 (8.8)
**Objective response rate** (**CR + ** **PR**)%	0.0
**Disease control rate** (**CR + ** **PR + ** **SD**)%	79.4

### Safety

Mean treatment duration was 7.5 months (range, 0.4 to 37.7). No patient discontinued treatment due to AEs. The most common AEs were stomatitis, anemia, and asthenia. The major Grade 3 and 4 AEs were asthenia (6%), infection (6%), and leukopenia (3%) (Table 3). The dose of everolimus was adjusted for 8 patients (24%). No unexpected toxicities of everolimus were observed.

**Table 3:** **Adverse events of any cause** (**reported in 10**% **or more of patients**, ***N*** = **34**)

Adverse events	All grades, ***N*** (%)	Grade 3 or 4, ***N*** (%)
Stomatitis	27 (79.4)	1 (2.9)
Anemia	22 (64.7)	1 (2.9)
Asthenia	13 (38.2)	2 (5.9)
Leukopenia	11 (32.4)	1 (2.9)
Neutropenia	10 (29.4)	0 (0.0)
Rash	10 (29.4)	0 (0.0)
Infection	8 (23.5)	2 (5.9)
Nausea	5 (14.7)	0 (0.0)
Dyspnea	5 (14.7)	0 (0.0)
Anorexia	5 (14.7)	0 (0.0)
Thrombocytopenia	4 (11.8)	0 (0.0)
Epistaxis	4 (11.8)	0 (0.0)
Cough	4 (11.8)	0 (0.0)

## Discussion

To the best of our knowledge, this study is the largest clinical trial of systemic treatment of advanced ACC. Although the primary hypothesis of this study was not fulfilled, everolimus had clinical efficacy in patients with ACC who all had disease progression before treatment. The median PFS duration was 11.2 months, 79.4% of patients achieved SD, and tumor shrinkage within SD criteria was observed in 44% of patients. Furthermore, pre- and post-treatment FDG-PET scans indicated a metabolic PR in 8 (44.4%) out of 18 evaluated patients. Interestingly, the length of PFS of the 8 patients with metabolic PR was longer than that of the other 10 patients with metabolic SD/PD. This survival difference suggests that an early metabolic response may be predictive of durable response.

The relative efficacy of everolimus observed in this study is more evident when it is compared with the efficacy of other molecular targeted agents reported in previous clinical trials. Because c-kit is expressed in a high proportion of ACCs [10, 11], imatinib, a c-kit tyrosine kinase inhibitor, was of potential interest. Two phase II studies examined the efficacy of imatinib in patients with ACC that had immunohistochemical evidence of c-kit expression [12, 13]. There was no objective response in the either study. In one study, SD was observed in 60% (9 of 15) of the patients, but median PFS duration was only 10 weeks [12]. The other study reported only 2 patients with SD among the 10 ACC patients [13]. Lapatinib, a dual inhibitor of EGFR and human epidermal growth factor receptor-2 (HER2) was studied in patients with EGFR and/or ErbB2 expressing ACC of the salivary gland [14]. That study, which included only patients with documented disease progression within 6 months of study entry, observed SD in 15 of 19 patients, but median PFS duration was only 3.5 months. A few other targeted agents showed promising efficacy, comparable to everolimus. EGFR inhibition by cetuximab resulted in SD in 20 of 23 patients and a median SD duration of 6 months [15]. Recently, a phase II study of sunitinib also achieved prolonged tumor stabilization, of >6 months, in 62% of patients with documented prior progression [16]. However, objective responses to targeted agents were rarely observed in patients with advanced ACC. Therefore, a novel combination of targeted agents could be a reasonable approach to improve the outcome of systemic treatment of advanced ACC.

## Conclusions

Everolimus showed a promising anti-tumor effect in the treatment of advanced ACC. Trials of novel combinations of everolimus with other targeted agents are warranted.
